# Improvement in Body’s Dynamic Adaptation during Walking with Vestibular Rehabilitation Therapy in Patients with Cerebellopontine Angle Tumor Resection

**DOI:** 10.3390/life14091100

**Published:** 2024-08-31

**Authors:** Natasa Kos, Tomaz Velnar, Marusa Brcar, Marko Brcar

**Affiliations:** 1 Medical Rehabilitation Unit, University Medical Centre Ljubljana, 1000 Ljubljana, Slovenia; natasa.kos@kclj.si (N.K.); marusa.brcar@kclj.si (M.B.); marko.brcar@kclj.si (M.B.); 2 AMEU-ECM Maribor, 2000 Maribor, Slovenia; 3 Clinical Department of Neurosurgery, University Medical Centre Ljubljana, 1000 Ljubljana, Slovenia

**Keywords:** surgery, balance impairment, vestibular rehabilitation therapy, functional gait assessment, gait

## Abstract

Background: Our study aimed to investigate the effects of vestibular rehabilitation therapy on functional gait performance in patients with balance disorders. Methods: A total of 40 post-operative patients with balance disorders were included in the study. They were divided into two groups and participated in a vestibular rehabilitation program during their hospital stay. After discharge, the intervention group performed vestibular exercises at home, while the control group did not. Balance was assessed using the Functional Gait Assessment Scale at discharge and three months after surgery. Results: The intervention group included 15 women and 5 men with an average age of 45 years, while the control group included 7 women and 13 men with an average age of 50 years. Three months after surgery, the change in Functional Gait Assessment (FGA) scores exceeded the clinically significant threshold of 5 points in 17 patients in the intervention group and 14 in the control group. There was a statistically significant difference in FGA progression between the groups (*p* = 0.034). After three months post-surgery, 7 patients in the intervention group experienced falls compared to 12 in the control group. Conclusion: Three months after surgery, we observed a significant improvement in the performance of balance tasks while walking and a lower risk of falls in the intervention group.

## 1. Introduction

After the surgical removal of a benign tumor in the cerebellopontine angle, patients often experience problems with their vestibular system. These can be severe dizziness, nausea, vomiting, headaches, and difficulty adapting to body movements during daily activities and walking. Patients often report feeling disorientated in space and have the sensation of spinning, swaying, or tilting [1]. After surgery, patients inevitably experience a loss of balance in their body and space due to conflicting sensory information from the visual, vestibular, and proprioceptive systems. These challenges undoubtedly have a negative impact on the well-being of patients of different age groups and genders [2]. In the early stages of recovery, patients often suffer from discomfort, limited motor skills, fear of falling, and psychological distress. These problems lead to functional limitations in basic tasks requiring postural balance, rapid rotation of the head and body in space, and good dynamic visual acuity [3]. Balance disorders significantly impair family life as well as professional and social activities and reduce the quality of life of those affected [4].

### 1.1. Assessment Tools in the Acute Phase

In hospital clinical practice, the patient’s essential functional independence is primarily assessed using the Barthel Index, which includes gait as a basic assessment aspect, including walking on level ground and stairs [5]. The Berg Balance Scale (BBS) is used to assess patients’ initial movement abilities by asking them to perform 14 everyday functional tasks. These tasks are structured to progress from the least demanding to the most demanding in terms of balance abilities, requiring the patient to focus on each activity [6,7]. Both measurement tools assess only part of the patient’s movement ability and do not cover all critical movement tasks required to perform activities of daily living and functional walking. In addition, they do not assess the patient’s intrinsic and extrinsic risk factors for falls, which require increased balance ability during functional gait. In the acute phase of recovery in hospital, objective assessment of a broader range of functional tasks during gait was essential. This included the assessment of functions from the simplest (for patients with severe to moderate balance impairments during walking) to the most demanding (for patients with mild impairments of dynamic balance during walking who are capable of more).

The Functional Gait Assessment (FGA) scale is useful to accurately monitor a patient’s dynamic gait adaptation, even in the early stages after surgery. It provides an objective and specific definition of the defective sensorimotor systems and enables the implementation of appropriate rehabilitation programs. If the rehabilitation programs are selected correctly and in time, they can significantly improve the patient’s balance abilities and skills [8]. The FGA scale assesses ten complex cognitive-motor tasks, including walking on a flat surface, walking with change of pace, walking with head rotation, walking with head tilt, walking and turning on the spot, crossing an obstacle, walking on a reduced support surface (tandem walking), walking with eyes closed, walking backward and climbing stairs [9]. Each FGA item is scored on a scale from 0 to 3. Grade 0 means severe impairment of the patient’s balance when walking, grade 1 means moderate impairment of balance, grade 2 means mild impairment, and grade 3 means no impairment of dynamic balance when walking [10]. A score below 22 out of 30 indicates a significant impairment of dynamic balance and an increased risk of falling, while a score above 22 indicates a mild impairment of dynamic balance with minimal risk of falling [11]. The FGA test takes 10 min to complete. In addition, the smallest clinically significant change in two FGA measurements that defines progress in functional gait is 5 points [12].

### 1.2. Acute Vestibular Rehabilitation Therapy

The primary goals of the initial vestibular rehabilitation therapy program for patients who have undergone surgical removal of a tumor in the cerebellopontine angle and suffer from balance disorders are to restore, improve, or maintain the ability efficiently to move in and out of bed, to move safely to higher sitting and standing positions, and most importantly, to maintain an upright posture and balance while walking and performing other functional tasks [13,14]. Treatment of patients with symptoms should initially include immediate and short-term bed rest, prompt replacement of fluids and electrolytes lost due to vomiting, and administration of medications to relieve symptoms, particularly nausea and vomiting. It is essential to keep this treatment as short as possible, e.g., one or two days to not interfere with the body’s compensatory mechanisms. Medications that support balance compensation include piracetam, amphetamines, extracts from the Ginkgo biloba tree, and caffeine [15].

Through early, individually tailored vestibular rehabilitation programs, we aim to directly influence balance compensation, i.e., the ability to spontaneously reactivate the central nervous system [16,17]. Vestibular rehabilitation is based on two primary mechanisms: adaptation and substitution. Adaptation means that the central nervous system adapts to the new state, which facilitates the rebalancing of neural activity and subsequently reduces responsiveness to disruptive stimuli. In substitution, alternative sensory systems are used to compensate for the absence of signals from the affected sensory systems. For example, if the organ of balance fails, visual perception can effectively replace its function. The individual adaptation of vestibular rehabilitation is an ongoing cognitive process that must be integrated into the patient’s recovery program immediately after surgery, with the simultaneous administration of appropriate pharmacological measures.

In this prospective study, we aimed to determine the effects of specific vestibular rehabilitation therapy on improving patients’ gait, assessed with an FGA scale before the hospital discharge and after three months post-surgery. Our aim was also to rigorously evaluate individual movement tasks during functional gait to definitively identify patients’ risk factors for falls.

## 2. Materials and Methods

### 2.1. Subjects

The study included 40 Slovenian-speaking patients admitted to the Clinical Department of Neurosurgery between January 2018 and June 2019 for planned surgical removal of a benign tumor in the cerebellopontine angle. Inclusion criteria included first-time surgery in the cerebellopontine angle, the ability to understand and follow instructions (minimum score of 25 out of 30 on the Mini-Mental State Examination (MMSE) [18]; the ability to walk 5 m with the help of a therapist or appropriate aid without undue fatigue, and a minimum score of 30 out of 56 on the Berg Balance Scale (BBS) to assess daily balance activities [7]. The evaluation was conducted based on the patient’s well-being immediately after the operation.

All participants gave their explicit, voluntary consent by signing a form. Patients under the age of 18 and over the age of 80, as well as patients with cognitive impairments or impaired consciousness, were excluded from participating in the study. The study was approved by the Ethics Committee of the Ministry of Health and strictly adhered to the principles of the Declaration of Helsinki on Biomedical Research Involving Human Subjects and the Slovenian Code of Medical Deontology (approval number 0120-472/2017/5, dated 2 November 2017).

### 2.2. Implementation

After surgical removal of a benign tumor from the cerebellopontine angle, patients were individually examined and treated in the intensive care unit of the Clinical Department of Neurosurgery at the University Medical Centre Ljubljana. In the first postoperative phase, a physician specializing in physical medicine and rehabilitation assessed the patients’ cognitive abilities using the MMSE [18]. After a thorough evaluation, we worked with a physiotherapist and an occupational therapist to assess the impaired sensorimotor systems that affect the patient’s limited ability to maintain balance while sitting, standing and walking. All patients suffered from post-operative dizziness, impaired vision, and eye movements and had varying degrees of difficulty actively adapting their bodies when lying down, sitting, standing, and especially during functional walking.

During the 21-day recovery period in the hospital, each patient took part in an individually tailored program of specific vestibular exercises. These exercises included tasks to stabilize vision, eye movements, head and body rotation in different positions and while walking. Before starting the vestibular exercises, it was essential to address the patients’ initial problems such as dizziness, nausea, and vomiting with appropriate medication. Once the primary symptoms were alleviated, patients gradually began to turn their heads in a reclined position, focusing on a target on the ceiling. To relieve the tension in the neck, we also introduced exercises focusing on rotating the shoulder girdle and stretching the neck muscles [19]. These exercises were performed three times a day, with each session lasting 5 to 15 min. At the beginning, the exercises were performed slowly and then gradually accelerated. It was found that the speed of compensation corresponded to the severity of dizziness and discomfort experienced during each vestibular exercise [20].

The fundamental components of vestibular training consist of deliberate physical training, exercises designed to alter sensory input, the center of gravity, or the support surface, and vestibular and functional exercises incorporated into walking routines [21].

According to the protocol of a randomized clinical study, the patients were randomly assigned to either the intervention or control groups after discharge from the hospital.

The intervention group consisting of 20 patients received a specially tailored program of vestibular exercises. They received comprehensive written and multimedia instructions on a USB stick and diaries to document their exercise performance and any falls. These tools were important to obtain feedback on the performance of the therapeutic exercises and the occurrence of possible falls.

Patients in the intervention group performed a variety of functional tasks while walking in different contexts and environments.

These exercises incorporated several risk factors to improve the patient’s balance skills during daily activities and walking. They were specifically designed to facilitate the transfer of the learned exercises from the hospital to the home environment, which is in line with the principles of motor learning. Vestibular training at home focused on improving the patient’s ability to maintain position and regulate reactive and proactive balance control during sitting, standing, and walking. This included adapting to a reduced support surface, adapting to changes in head and body alignment, performing various upper limb tasks, and coping with reduced proprioceptive input. Patients in the control group were only given a diary to document any falls and were given verbal instructions for vestibular exercises on discharge to their home environment. We assessed patients’ walking ability using the FGA scale before discharge from the hospital and three months after surgery.

We used Microsoft Excel 2019 from Microsoft Corp, Redmond, WA, USA 2019, and the IBM SPSS Statistics 27 program for the Windows environment to effectively collect and analyze data and create visual representations. We rigorously calculated descriptive statistics for the variables under consideration for both patient groups, including mean, median, interquartile range, and standard deviation.

We performed a mixed repeated-measures analysis of variance (ANOVA) to compare the development of numerical variables between the intervention and control group. In addition, we used Pearson’s correlation coefficient to reliably assess the relationship between the variables. According to the relevant literature [12], a change of 5 points in the FGA measures was clinically significant. Our threshold for statistical significance was set at *p* = 0.05. Furthermore, we rigorously confirmed that all assumptions of the analysis of variance were consistently met, including distribution, homogeneity, independence, variance, and error values.

## 3. Results

A total of 40 patients, 55% women and 45% men, ranging in age from 18 to 75 years and with a mean age of 47 years, actively participated in the study. Of these, 20 patients (75% women and 25% men) with an average age of 45 years belonging to the intervention group. In comparison, the remaining 20 patients (35% women and 65% men) with an average age of 50 years participated in the control group. During the 21-day recovery period in the hospital, all participants experienced vestibular disturbances when sitting, standing, and walking. Table 1 shows the 95% confidence interval for FGA and the effect size for the difference between groups in progression for FGA: Cohen’s d of 0.71 (95% CI 0.063–1.34). Table 2 shows the number of patients in the intervention group and the control group who required supervision, therapist help, or various walking aids during the safe walking assessment, as well as the number of falls patients experienced before hospital discharge and three months after surgery.

**Figure 1:**
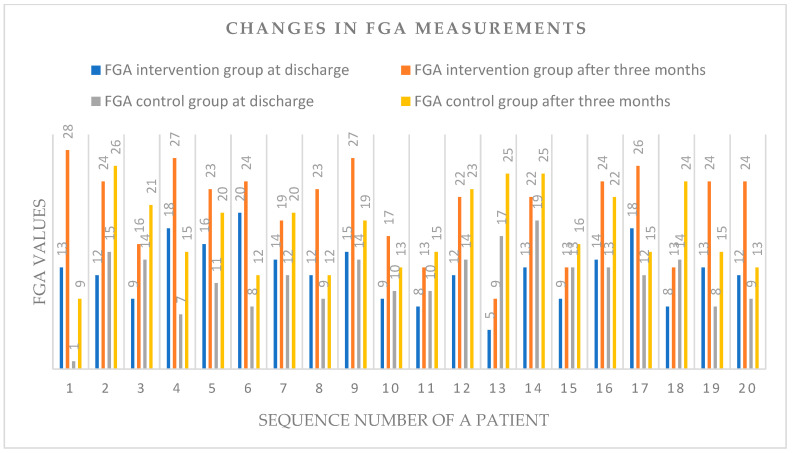
Graphical distribution of changes in FGA measurements for all patients from both groups before hospital discharge and three months after surgery.

Table 3 displays individual scores ranging from 0 to 3 for dynamic postural adaptation for patients in the intervention group and the control group while performing specific movement tasks after FGA, before discharge from the hospital to the home environment, and three months after surgery. Figure 1 shows the graphical representation of the changes in the FGA measurements for all patients from both groups before hospital discharge and three months after surgery.

After being discharged home, all patients had severe to moderate balance disorders, with FGA scores of less than 22 out of 30. After surgery, we performed a follow-up examination after three months to assess the patient’s walking ability using the FGA scale. Of the patients who participated in the intervention, 65% improved their dynamic balance by scoring more than 22 out of 30. Conversely, 35% of the patients in the training group did not reach the threshold of 22/30 points. In the control group, 35% of patients achieved more than 22/30 points in reassessing functional walking with FGA after three months, while 65% did not reach 22 points. It is worth noting that dynamic balance in the control group improved by an average of 15 points, although most patients did not exceed 22 points on the FGA scale.

The FGA scores exhibited a statistically significant variance between the groups (*p* = 0.034). Furthermore, a strong correlation (r = 0.852; *p* < 0.001) was identified between the scores after patients’ discharge to their home environment and their scores three months post-surgery.

To account for the significant difference between genders in our sample, we performed a sensitivity analysis with a two-way analysis of covariance (ANCOVA). This rigorous analysis considered the group effects and the influence of age and gender on progress after the FGA. Remarkably, the group effect remained statistically significant in this analysis.

The smallest clinically significant change in two FGA measurements that defines progress in functional gait is 5 points [12]. Three months after surgery, the change in the Functional Gait Assessment (FGA) scores exceeded the clinically significant threshold of 5 points in 17 patients in the intervention group and 14 in the control group.

On discharge from the hospital to their home environment, all patients from both groups were dependent on external assistance and crutches and unable to walk independently and functionally. Three months later, a retest showed that 65% of patients in the intervention group could walk independently, compared to 40% of patients in the control group.

After discharge from the hospital and return home, all patients were at risk of falling (100%), but the risk decreased after three months. At the three-month follow-up examination, it was found that there were differences in the risk of falling between the two groups. In total, 7 patients in the intervention group fell, while 12 patients in the control group fell. At discharge, 5–35% of patients were independent in level walking, stair climbing, and turning. However, all patients had moderate to severe dynamic balance impairments and were not independent in activities such as variable speed walking, tandem walking, backward walking, walking with eyes closed, walking over obstacles, and walking with head movements in horizontal and vertical planes that required increased proprioceptive and vestibular input.

Analysis of the individual movement tasks based on the FGA shows that three months after surgery, most patients in both the intervention and control groups had achieved independence in walking on level ground, turning, and climbing stairs. However, it should be noted that not all patients in both groups achieved independence when walking with their eyes closed or with their head turned horizontally.

In the intervention group, around 50% of patients were able to independently perform challenging walking exercises such as changing speed, walking over obstacles, vertical head tilt, walking backward, and tandem walking. In the control group, 50% of patients were able to walk independently with vertical head turns. However, only 10–25% of patients were independent in walking backward, changing speeds, crossing obstacles, and tandem walking.

In the intervention group, 75% of patients showed mild impairments in dynamic body adaptation while walking with the head turned horizontally, and 25% showed impairments with eyes closed. In the control group, 50% of patients showed mild impairments in dynamic balance while walking with the head turned horizontally, and 40% had difficulty walking over obstacles. Between 20 and 35% of patients in both groups had moderate to severe problems with dynamic posture when walking backward, walking with the head turned horizontally and walking over obstacles. In addition, 75% of patients in the intervention group had difficulty walking with their eyes closed. In the control group, most patients (95%) refrained from walking with their eyes closed, 65% could not walk independently in tandem, and 50% had problems walking over obstacles, walking backward, and walking with their head turned horizontally.

## 4. Discussion

The hospital’s rehabilitation team aims to accelerate the improvement in vestibular compensation in patients suffering from balance disorders after the surgical removal of a benign tumor in the cerebellopontine angle. This goal is achieved by the immediate implementation of individually customized vestibular rehabilitation programs. By systematically introducing repetitive movements of the eyes, head, and body in sitting and standing positions, especially during walking, in conjunction with the integration of daily intermediate movements, we aim to assess the patient’s dynamic postural control during walking and their ability to adapt to potentially fall-inducing factors from the intrinsic or extrinsic environment.

In the acute recovery period, the patient must maintain proper head and trunk orientation concerning gravity to effectively control balance and movement at high speeds and frequencies. Sensory references are pivotal in postural control during this critical phase [22].

In recent decades, significant advances have been made in vestibular rehabilitation techniques to improve adaptation to loss of balance, habituation to altered sensory conditions and adjustment of sensory weighting. The effectiveness of these techniques relies heavily on the qualifications of the rehabilitation team. We recommend that the team consist of physiatrists, psychologists, otolaryngologists, neurologists, physiotherapists, and occupational therapists with a minimum of five years of experience treating neurological patients. Additionally, therapists should have completed postgraduate professional development in vestibular rehabilitation.

The individual assessment of dysfunctional sensorimotor systems and the implementation of specific, targeted therapeutic programs for their treatment is still uncommon. In hospitals, vestibular rehabilitation is often applied to patients in a non-specific manner, regardless of the clinical findings. However, it is essential to note that even with this non-specific approach, vestibular rehabilitation is considered a safe option for early, targeted therapeutic intervention. It is risk and side effect free, which provides peace of mind for both patients and healthcare professionals. It is also a cost-effective solution, making it a practical choice in healthcare. In addition, it encourages repetitive movements of the eyes, head, and trunk. In combination with traditional physiotherapy such as manual therapy of the cervical spine and balance and proprioception exercises, it shows positive effects soon after surgery. This shows the importance of developing more challenging exercises for patients [23].

During the hospital stay, all patients completed the vestibular rehabilitation program.

We carefully ensured a safe environment and provided them with the necessary aids or physical support to perform various balance exercises. The exercise program was individually tailored to each patient, depending on their well-being, motivation and fatigue. After discharge to their home environment, the intervention group received a comprehensive balance exercise program and a protocol for the exercises in written form on a USB stick. The vestibular training program was specifically targeted at the autonomic phase of motor learning and focused on maintaining position and regulating proactive and reactive control of sitting, standing and walking postures. In contrast, the control group, who received no movement training after discharge, received only verbal instructions on how to perform balance exercises and a diary in which all falls were recorded.

Our study clearly shows that the intervention group and the control group, regardless of their exercise activity before discharge, showed significant and clinical meaningful improvements in walking ability three months after surgery, as assessed by the FGA scale. In particular, there was a statistically significant divergence in the development of FGA scores between the groups (*p* = 0.034). Severe or moderate impairment of dynamic balance is present when the majority of tasks are rated 0 or 1 on the FGA scale or when the total FGA score falls below 22 out of 30. Conversely, mild or no impairment is present if the total FGA score is above 22 and most tasks are rated 2 or 3 [9]. After a three-month follow-up examination, there were differences in the risk of falling between the two groups. In the intervention group, 7 patients fell, while 12 patients in the control group experienced a fall. According to Wrisley and colleagues’ evaluation of healthy older people, a total FGA score of less than 22 points indicates an increased risk of falling and the presence of dynamic balance disorders. They accurately predicted six out of seven falls [10].

None of the patients in the intervention group and the control group were able to perform all 10 FGA movement tasks independently while walking. These tasks were evaluated using a 4-point scale for assessing walking functionality, the FGA scale, which ranges from 0 to 3.

In our study of 40 patients, we found that specific locomotor tasks are more common in the daily lives of patients in hospitals and at home. Tasks such as walking on a flat surface, climbing stairs, and walking with turns are generally less demanding, while functions such as walking with eyes closed and walking with head turned require sharper balance skills and are more challenging. In our intervention group, we found a higher representation of movement tasks such as walking with speed change, walking with obstacles, walking backward and walking with head rotation in the vertical plane compared to the control group. The difference in representation was 5–40 percentage points (Table 3)

The correlation between this finding and the patients’ impaired sensorimotor systems is clear. Walking in tandem, walking backward, walking with eyes closed, and walking over obstacles all require increased input from the somatosensory system. In addition, walking with head tilt and rotation in both the vertical and horizontal planes and walking with changes in speed requiring increased responsiveness of the vestibulospinal and vestibulocochlear systems, which are inevitably impaired to some degree in all patients who have undergone cerebellopontine angle surgery.

The results of our study stand alone and cannot be compared with other studies as there has been no established analysis of FGA movement tasks in the acute recovery phase in previous studies.

Our FGA analysis showed that the intervention group significantly improved their balance three months post-surgery by performing customized balance exercises in their home environment. These exercises effectively addressed various risk factors and enhanced proactive and reactive balance responses in different contexts and environments.

A thorough literature review by a group of authors concluded that vestibular rehabilitation plays a central role in balance compensation through key mechanisms of neuroplasticity. Ultimately, these exercises alleviate or eliminate the patient’s balance symptoms [24,25].

The process of recovery from vestibular problems differs between static deficits (body and head tilt) and dynamic deficits (balance problems during movement). The compensation of static deficits takes place over several days to weeks through neuroplasticity in the vestibular nuclei of the brainstem. The compensation of dynamic deficits, on the other hand, is incomplete and protracted and requires adaptation and sensorimotor replacement by the central nervous system. We can help improve these problems by increasing the impulses in the central vestibular balance pathways through specific vestibular exercises [26].

Given the proven benefits of vestibular rehabilitation, future research must address certain limitations in this study. Specifically, a larger sample size of patients with the same diagnosis is necessary to achieve better results. It is crucial to improve the randomization of patients by gender and age, especially considering the age difference, as older individuals are more likely to struggle with balance and have an increased risk of falls. In addition, our study did not include patients’ medication history after discharge and three months post-surgery, so this has to be considered in subsequent analyses. Furthermore, conducting professional monitoring of vestibular balance exercises at home via video links is essential to gather more data on how personalized vestibular training can reduce the risk of falls and improve rehabilitation progress.

## 5. Conclusions

Our study found that the inclusion of repetitive eye, head, and trunk movements during walking resulted in clinical and statistical improvement in gait performance in each patient during the recovery period in the hospital and three months after surgery. In addition, we observed that individualized vestibular training had a positive effect on movement tasks requiring improved balance ability during walking, thereby reducing the risk of falling. Vestibular rehabilitation in hospitals is a safe and cost-effective practice for patients with balance disorders. However, its use in the assessment of impaired sensorimotor systems and the formulation of early individualized therapeutic programs is currently insufficient and non-specific. Despite the positive impact of the research, further studies with a larger sample of patients is essential, and the age difference must also be considered, as older individuals are more likely to struggle with balance and have an increased risk of falls.

In addition, our study also did not include patients’ medication history after discharge and three months post-surgery, so this has to be considered in subsequent analyses. We strongly believe that a professionally qualified rehabilitation group should be established for future research. This group would focus on assessing impaired sensorimotor systems, developing targeted individualized vestibular therapy programs involving complex movement tasks, and closely monitoring patients’ rehabilitation progress over time.

## Figures and Tables

**Table 1:** The 95% CI for FGA and effect size for between-group FGA differences.

		n1	n2
FGA at discharge	mean (95% CI)	11.95	11.5
	Min.	9.81	9.64
	Max.	14.09	13.36
FGA after three months	mean (95% CI)	21.25	18.8
	Min.	18.54	16.35
	Max.	23.96	21.25
FGA progress	mean (95% CI)	9.3	7.3
	Min.	7.77	6.21
	Max.	10.83	8.39

Legend: n1—20 patients in the intervention group; n2—20 patients in the control group. CI—confidence interval, FGA—functional gait assessment

**Table 2:** Use of walking aids, supervision, and assistance from a therapist or other person in assessing patients and the number of falls after hospital discharge and three months after surgery.

Walking Aid	After Discharge		Three Months after Surgery	
	n1 %	n2 %	n1 %	n2 %
The stilt and the therapist	8 40%	10 50%	1 5%	1 5%
Stilt and control	7 35%	8 40%	3 15%	2 10%
Stilt	/	/	/	5 25%
Therapist	3 15%	1 5%	/	/
Control	2 10%	1 5%	3 15%	4 20%
No walking aid	/	/	13 65%	8 40%
FALLS				
YES	0 0%	0 0%	7 35%	12 60%
NO	20 100%	20 100%	13 65%	8 40%

Legend: n1—20 patients in the intervention group; n2—20 patients in the control group.

**Table 3:** Individual evaluations (rated from 0 to 3) of dynamic postural adjustment in the intervention group and control group during individual FGA movement tasks before discharge from the hospital to the home environment, and three months after the operation.

Motor FGA Tasks	After Hospital Discharge								Three Months after Surgery							
	**3**		**2**		**1**		**0**		**3**		**2**		**1**		**0**	
	n1	n2	n1	n2	n1	n2	n1	n2	n1	n2	n1	n2	n1	n2	n1	n2
Walking on the level	7 35%	4 20%	12 60%	15 75%	1 5%	1 5%	/	/	18 90%	19 95%	2 10%	1 5%	/	/	/	/
Walking with a change of speed	3 15%	/	8 40%	3 15%	9 45%	16 80%	/	1 5%	11 55%	4 20%	8 40%	15 75%	1 5%	1 5%	/	/
Walking with head turning left/right	/	/	1 5%	2 10%	12 60%	12 60%	7 35%	6 30%	/	/	15 75%	10 50%	5 25%	8 40%	/	2 10%
Walking with head tilts up/down.	/	/	9 45%	14 70%	8 40%	4 20%	3 15%	2 10%	9 45%	9 45%	11 55%	6 30%	/	5 25%	/	/
Walking and turning	4 20%	4 20%	14 70%	13 65%	1 5%	2 10%	1 5%	1 5%	15 75%	16 80%	4 20%	4 20%	1 5%	/	/	/
Walking over obstacles	/	/	7 35%	7 35%	8 40%	9 45%	5 25%	4 20%	10 50%	2 10%	3 15%	8 40%	5 25%	5 25%	2 10%	5 25%
Walking backwards	/	/	4 20%	7 35%	10 50%	8 40%	6 30%	5 25%	8 40%	5 25%	8 40%	6 30%	4 20%	8 40%	/	1 5%
Walking with eyes closed	/	/	/	/	2 10%	2 10%	18 90%	18 90%	/	/	5 25%	1 5%	5 25%	7 35%	10 50%	12 60%
Tandem walking	/	/	/	/	4 20%	5 25%	16 80%	15 75%	7 35%	3 15%	8 45%	4 20%	5 25%	5 25%	/	8 40%
Walking up the stairs	3 15%	1 5%	16 80%	18 90%	/	/	1 5%	1 5%	13 65%	12 60%	7 35%	8 45%	/	/	/	/

Legend: n1—20 patients in the intervention group; n2—20 patients in the control group; 0—severe impairment of dynamic balance. 1—moderate impairment of dynamic balance. 2—mild impairment of dynamic balance. 3—no impairment of dynamic balance.

## Data Availability

The original contributions presented in the study are included in the article, further inquiries can be directed to the corresponding author.
